# Association of the Lipidome With Alzheimer's Disease and the Mediated Effect of Metabolites: A Two‐Step Mendelian Randomization Study

**DOI:** 10.1002/brb3.70352

**Published:** 2025-02-19

**Authors:** Yunfeng Yu, Juan Deng, Xinyu Yang, Jingyi Wu, Rong Yu, Chenlu Guo

**Affiliations:** ^1^ The First Hospital of Hunan University of Chinese Medicine Changsha Hunan China; ^2^ School of Traditional Chinese Medicine Hunan University of Chinese Medicine Changsha Hunan China; ^3^ The Third School of Clinical Medicine Zhejiang Chinese Medical University Hangzhou Zhejiang China

**Keywords:** Alzheimer's disease, lipidome, metabolites, mediated effect, Mendelian randomization

## Abstract

**Objective:**

This study aimed to explore the causal effects of lipidome on Alzheimer's disease (AD) and the mediated effects of the metabolites using Mendelian randomization (MR).

**Methods:**

Data were obtained in genome‐wide association studies, and single‐nucleotide polymorphisms were screened according to the underlying assumptions of MR. Subsequently, weighted inverse variance was used to analyze the causality of lipidome with AD as well as the mediated effects of metabolites. Finally, MR‐Egger, Cochran's *Q*, and sensitivity analysis were used to assess horizontal pleiotropy, heterogeneity, and robustness of the results, respectively.

**Results:**

The MR analysis showed that phosphatidylcholine (PC) (15:0_18:2) (mediated proportion: 18.30%, *p* = 0.024) and phosphatidylethanolamine (PE) (18:0_18:2) (mediated proportion: 14.60%, *p *= 0.028) mediated the reduction of AD risk by lowering betaine levels, which revealed lipidomic susceptibility. The MR‐Egger intercept showed no horizontal pleiotropy for all results (*p *≥ 0.05). Cochran's *Q* showed heterogeneity in some of the results. Sensitivity analysis indicated that all results were robust.

**Conclusion:**

Our findings reveal the pathways through which PC (15:0_18:2) and PE (18:0_18:2) mediated the reduction of AD risk by lowering betaine levels.

## Graphical abstract

The Mendelian randomization analysis suggested that phosphatidylcholine (15:0_18:2) and phosphatidylethanolamine (18:0_18:2) reduced genetic susceptibility to Alzheimer's disease (AD) by lowering betaine levels. This finding offers new genetic insights into understanding the pathogenesis and developing novel drugs for AD.

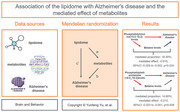

**Abbreviations**
AD: Alzheimer's disease
APP: β‐amyloid precursor protein
CI: confidence interval
GWAS: genome‐wide association studies
IL: interleukin
MR: Mendelian randomization
OR: odds ratio
PC: phosphatidylcholine
PE: phosphatidylethanolamine
SNP: single‐nucleotide polymorphism

## 1 Introduction

Alzheimer's disease (AD) is one of the most prevalent progressive neurodegenerative diseases, which is characterized by memory loss, cognitive dysfunction, behavioral abnormalities, and social impairments (Dzamba et al. 2016). According to the International Alzheimer's Disease Organization, approximately 50 million people worldwide had dementia in 2018, and it is expected to triple by 2050 (World Alzheimer Report 2018). AD is the leading cause of dementia, accounting for 60%–80% of dementia cases globally (Rostagno 2022). It not only affects the physical and mental health of patients and caregivers but also imposes a significant medical and economic burden on families and society (Alzheimer's Association 2023). According to the 2023 Alzheimer's Disease Facts and Figures report, AD has been officially listed as the sixth leading cause of death in the United States (Alzheimer's Association 2023). In the United States alone, there are 6.7 million people over the age of 65 with AD, and this number is expected to increase to 13.8 million by 2060 (Alzheimer's Association 2023). Currently, drugs such as donepezil, memantine, galantamine, and rivastigmine, which are approved for marketing by the Food and Drug Administration, are the primary treatment options for AD (Breijyeh and Karaman 2020). However, they only stabilized or improved cognition and activities of daily living in patients with AD without addressing the root cause of AD (Yiannopoulou and Papageorgiou 2020). Moreover, constrained by the permeability of the blood‐brain barrier, the effects of regular doses of drugs are limited, while escalating doses may engender a cascade of secondary adverse events (Zenaro et al. 2017; Chakraborty et al. 2017). Unfortunately, although the abnormal accumulation of β‐amyloid in the brain is recognized to be a key mechanism for developing AD, and specific drugs targeting this mechanism have been developed, such as lecanemab and aducanumab, these therapies have shown limited clinical efficacy in halting or reversing disease progression (Weller and Budson 2018; van Dyck et al. 2023; Knopman et al. 2021). Therefore, we need to explore and understand the pathogenesis of AD from different perspectives and develop novel drugs for the prevention or treatment of AD on this foundation.

Lipids, constituting 60% of the dry weight of brain tissue, are among the most crucial components, which are involved in functions such as the construction of nerve cell membranes, nerve conduction, and cell signaling (Kalli 2020). Recent studies have highlighted the significance of changes in brain lipid content and composition induced by environmental factors as pivotal contributors to the development of AD (Chiurchiù et al. 2022). Lipids have been reported to be involved in the transport and processing of β‐amyloid precursor protein (APP) and to influence the synthesis of β‐amyloid (Penke et al. 2018). β‐Amyloid is a neurotoxic one‐way transmembrane protein, and its aberrant accumulation in the brain is considered to be a key factor in the development of AD (O'Brien and Wong 2011). Previous studies have also identified abnormal lipid metabolism in neuroglia cells of AD brains (Haney et al. 2024) and suggested that lipids influence AD through multiple metabolic pathways (Zarrouk et al. 2018). Subsequent studies have shown that disturbed lipid metabolism affects neuronal membrane stability and triggers neuroinflammation, ultimately leading to neuronal damage and loss of synaptic connectivity (Roy and Tedeschi 2021; Olsen and Færgeman 2017; Zhou et al. 2023). Although early cross‐sectional studies explored the relationship between lipids and AD, they could not explain the causal relationship between the two and were subject to potential confounding factors. Therefore, we need to explore an effective and comprehensive approach to further assess the causal effects of the lipidome and its metabolites on AD.

Mendelian randomization (MR) is a widely used analytical method in epidemiological studies that infers causal relationships between exposures and outcomes using single‐nucleotide polymorphisms (SNPs) (Bowden and Holmes 2019). Since genetic variation follows Mendel's first law during meiosis, MR minimizes confounding factors and reverses causality (Emdin et al. 2017). In this study, we used two‐sample MR to assess the causal effects of lipidome on AD and the mediated effects of metabolites.

## 2 Materials and Methods

### 2.1 Study Design

**FIGURE 1:**
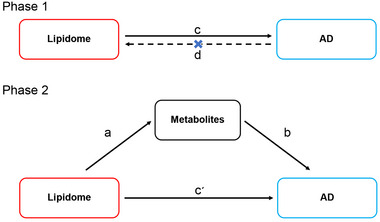
MR design diagrams of lipidome–metabolites–AD. The a is the effect of lipidome on metabolites; the b is the effect of metabolites on AD; the c is the total effect of AD; the cʹ is the direct effect of lipidome on AD; and the d is the total effect of AD on lipidome. The algorithm for the mediated effect is a × b. The algorithm for the mediated proportion is (a × b)/c. AD, Alzheimer's disease.

This MR study analyzed the effect of lipidome on the risk of AD and the mediated effects of metabolites, which was reported according to the Strengthening the Reporting of Observational Studies in Epidemiology Using Mendelian Randomization (Skrivankova et al. 2021). Specifically, we analyzed the effect of genetic variants related to lipids on AD risk and metabolite levels, as well as the effect of genetic variants related to metabolites on AD risk. It consisted of two phases, as shown in Figure 1. Phase 1: Assessed the causal relationship between lipidome and AD using bidirectional MR. It required a significant causal effect of lipidome on AD and no significant effect of AD on lipidome. Phase 2: Assessed the mediated effects of metabolites in the effect of lipidome on AD. It required that the lipidome has a significant causal effect on metabolites and that metabolites have a significant causal effect on AD.

### 2.2 Data Sources

Data for the lipidome were derived from the GeneRISK cohort (Ottensmann et al. 2023). The cohort recruited 7174 Finns, aged 45–66 years, in southern Finland between 2015 and 2017. It randomly invited 4857 individuals through population registers and recruited 1369 individuals and 1116 blood donors through private occupational healthcare organizations and advertisements, respectively. Of these, individuals under guardianship, those with a history of atherosclerotic cardiovascular disease, and pregnant women were excluded from the study. Before the collection of blood samples, participants were asked to fast overnight for 10 h. Subsequently, participants' blood samples were collected for plasma, serum, and DNA extractions and ultimately compiled into a GWAS of 179 lipid species in 13 lipid classes.

Data for metabolites were obtained from the Canadian Longitudinal Study on Aging cohort (Chen et al. 2023). The cohort followed more than 50,000 participants aged 45–85 years and ultimately included 8299 unrelated European subjects. After collecting plasma samples from the participants, Metabolon HD4 was used to analyze the plasma samples for 1458 metabolites and ultimately compiled 1091 metabolites and 309 metabolite ratios for GWAS.

Data for AD were obtained from FinnGen (dataset number: finngen_R10_G6_ALZHEIMER) (Kurki et al. 2023). FinnGen has aggregated samples from the nationwide network of Finnish Biobank and digital healthcare data from the National Institute for Health and Welfare and combined pseudonymized registry data with the minimal phenotypic dataset from the Finnish Biobank. AD is defined by FinnGen as a progressive neurodegenerative disorder characterized by the loss and death of neurons in multiple regions of the brain, leading to cognitive impairments such as memory and language deficits. It is diagnosed through medical history, cognitive function assessment, brain imaging examinations (MRI, CT, PET), and biomarker testing (β‐amyloid and Tau protein levels). It provides GWAS data for 412,181 Europeans, including 10,520 cases in the experimental group and 401,661 cases in the control group. As these data are publicly available, additional ethical approval is no longer required. Detailed sources of exposure and outcome data are provided in Table 1.

**TABLE 1:** Detailed description of exposure and outcome phenotypes.

Phenotype	PMID	Population	Sample size	Definition
Lipidome	37907536	European	7174	Mass spectrometry‐based lipid analysis was performed for 7302 individuals from the GeneRISK cohort by shotgun lipidomic analysis at Lipotype GmbH (Dresden, Germany). The analysis was performed by direct infusion in a QExactive mass spectrometer from Thermo Scientific with a TriVersa NanoMate ion source from Advion Biosciences. The lipidomics data were analyzed using lipid identification software and a data management system developed in‐house by Lipotype Gmb. The lipids with a high signal‐to‐noise ratio (> 5) and amounts at least fivefold higher than the corresponding blank samples were included. By including eight reference samples per 96‐well plate batch, reproducibility was assessed, and the lipid amounts were corrected for batch variations and analytical drift if the *p* value of the slope was < 0.05 with an *R* ^2^ > 0.75 and the relative drift > 5%. The lipid species detected in more than 70% of the samples were included (179 lipid species from 13 lipid classes). After the samples with a very low total lipid content and with > 30% of the 179 lipids missing were excluded (*N* = 26), data from 7276 individuals remained
Metabolites	36635386	European	8299	Of the 1091 plasma metabolites tested, 850 had known identities across eight super pathways (i.e., lipid, amino acid, xenobiotics, nucleotide, cofactor and vitamins, carbohydrate, peptide, and energy). The remaining 241 were categorized as unknown or “partially” characterized molecules. The levels of 1458 metabolites were quantified in plasma samples by Metabolon Inc. (Durham, NC, USA) using the ultrahigh performance liquid chromatography–tandem mass spectroscopy (UPLC‐MS/MS) platform, which is also known as the Metabolon HD4 platform. Strict QC and curation of the metabolomics data were applied to ensure accurate and consistent identification of true chemical entities and to remove those representing systemic artifacts, misassignments, and background noise. They then used batch‐normalized levels of metabolites generated by the Metabolon and only retained metabolites that had missing measurements in fewer than 50% of samples (*N* = 1091)
Alzheimer's disease	36653562	European	412,181	Pseudonymized register data were combined with the minimum phenotype dataset from the Finnish biobanks (age, sex, year of sampling, height, weight, and smoking status). Clinical end points were constructed from the register codes using the Finnish version of the International Classification of Diseases, 10th revision (ICD‐10) diagnosis codes and harmonizing those with definitions from ICD‐8 and ICD‐9. Alzheimer's disease: A progressive, neurodegenerative disease characterized by loss of function and death of nerve cells in several areas of the brain, leading to loss of cognitive function such as memory and language

### 2.3 Selection of Genetic Instrumental Variables

SNPs are the most common form of genetic variants in the genome. We identified the relevant SNPs for causal analysis of the lipidome on AD, AD on the lipidome, the lipidome on metabolites, and metabolites on AD through the following five steps. First, a significance threshold of *p* < 5 × 10^−5^ was used to identify SNPs closely associated with the lipidome and metabolites, and a more stringent threshold of *p* < 5 × 10^−8^ was applied to identify SNPs related to AD. Second, SNPs within kb = 10,000 and *R* ^2^ < 0.001 were selected to ensure independence and avoid linkage disequilibrium. Third, SNPs highly correlated (*F* > 0) were retained to eliminate weakly correlated variables. The calculation of *F* was defined as *F* = [*R* ^2^/(1 − *R* ^2^)] × [(*N* − *K* − 1)/*K*]. *K* refers to the number of paired samples, *N* refers to the total number of samples, and *R* ^2^ refers to the cumulative explained variance. Fourth, palindromic and ambiguous SNPs were removed when adjusting allelic orientations of exposures and outcomes. Fifth, outlier SNPs (*p *< 1) were excluded during the MR‐Pleiotropy RESidual Sum and Outlier‐based correction analysis to uphold the causal validity of the inference.

### 2.4 Data Analysis

After determining the SNPs of each group of exposure‐outcome, we used a two‐step MR study to analyze the effect of lipidome on the risk of AD and the mediated effects of metabolites. First, two‐sample MR was used to analyze the bidirectional causality between lipidome and AD. It aimed to identify the lipidome that has a significant effect on AD and was not interfered by reverse causality and to obtain the total effect of the lipidome on AD (c). Second, two‐sample MR was used to further analyze the causal effects of lipidome on metabolites (a) and metabolites on AD (b) to find the lipidome and metabolites that satisfy the “lipidome–metabolites–AD” pathway. Third, the mediated effect and mediated proportion of metabolites in the action of the lipidome to AD were calculated based on the above data. The mediation effect = a × b, and the confidence interval (CI) was calculated by the delta method. Mediated proportion = (a × b)/c. The above analyses were performed by the R 4.3.1 software with the TwoSampleMR (0.5.7) package installed. Weighted inverse variance, a method that enabled unbiased analysis in the absence of pleiotropy, was set up as the main tool for MR analysis. In addition, weighted median, an analytical method sensitive to outliers, and MR‐Egger, a method to analyze data in the presence of pleiotropy, were set as secondary tools for MR analysis. MR‐Egger was also used for assessing horizontal pleiotropy, with results indicating no horizontal pleiotropy when *p* ≥ 0.05. Cochran's *Q* and the leave‐one‐out method were used for the heterogeneity analysis and sensitivity analysis of MR results. There was no heterogeneity in the results when *p* ≥ 0.05, and the results were robust when the combined effect sizes were not significantly altered.

## 3 Results

### 3.1 Genetic Instrumental Variables

The MR analysis reported two pathways related to “lipidome–metabolites–AD.” It contained two causal effects of “lipidome–metabolites,” one causal effect of “metabolites–AD,” and two causal effects of “lipidome–AD.” The relevant SNPs are shown in Tables S1–S5.

### 3.2 Lipidome–Metabolites

**FIGURE 2:**
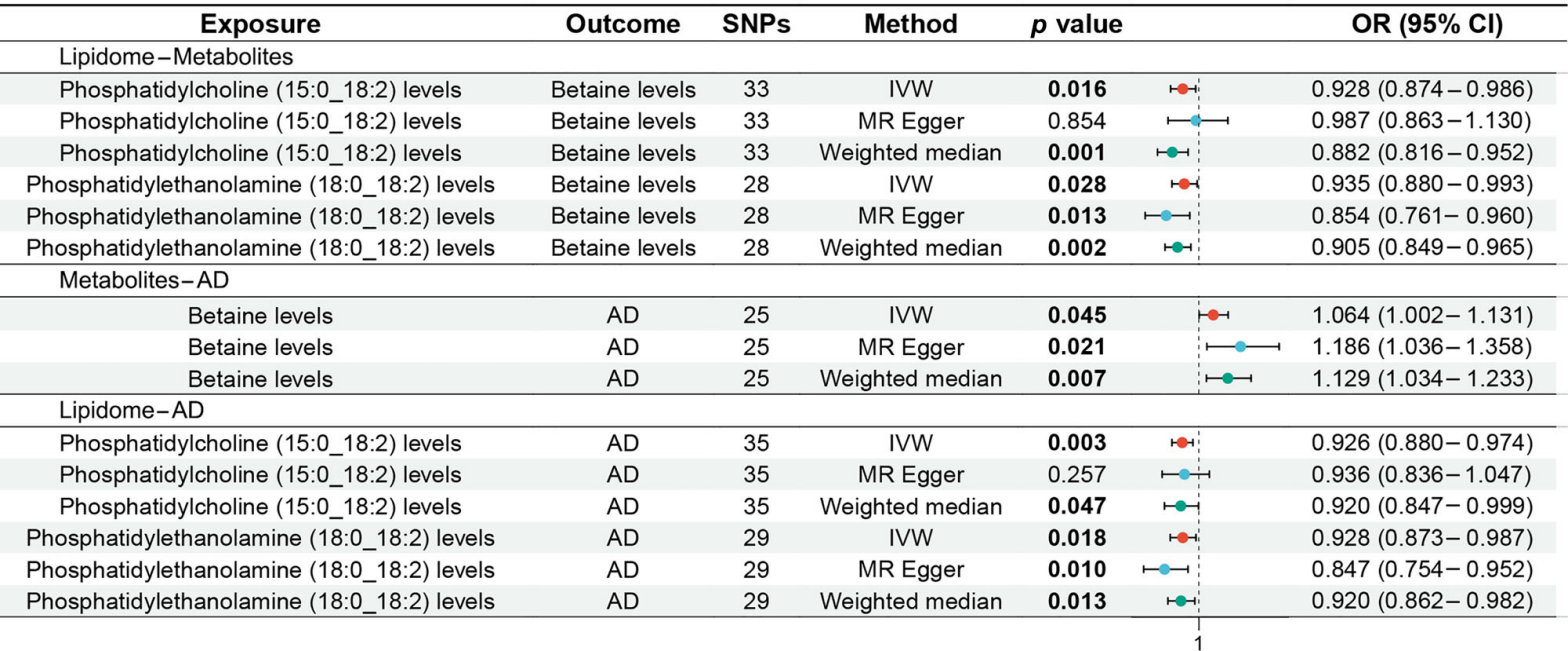
Forest plot of MR analysis for lipidome–metabolites–AD. MR, mendelian randomization. AD, Alzheimer's disease.

The MR analysis showed that phosphatidylcholine (PC) (15:0_18:2) levels (odds ratio [OR]: 0.928, 95% CI: 0.874–0.986, *p *= 0.016) and phosphatidylethanolamine (PE) (18:0_18:2) levels (OR: 0.935, 95% CI: 0.880–0.993, *p *= 0.028) were associated with reduced betaine levels, as shown in Figure 2 for the forest plot and Figure S1A,B for the scatter plot. MR‐Egger showed no horizontal pleiotropy in the results (*p *≥ 0.05), as shown in Table S6.

### 3.3 Metabolites–AD

The MR analysis showed that betaine levels were associated with the increased risk of AD (OR: 1.064, 95% CI: 1.002–1.131, *p *= 0.045), as shown in Figure 2 for the forest plot and Figure S1C for the scatter plot. MR‐Egger showed no horizontal pleiotropy in the results (*p *= 0.092), as shown in Table S6.

### 3.4 Lipidome–AD

The MR analysis revealed that PC (15:0_18:2) levels (OR: 0.926, 95% CI: 0.880–0.974, *p *= 0.003) and PE (18:0_18:2) levels (OR: 0.928, 95% CI: 0.873–0.987, *p *= 0.018) were associated with the reduced risk of AD, as shown in Figure 2 for the forest plot and Figure S1D,E for the scatter plot. MR‐Egger showed no horizontal pleiotropy in the results (*p *≥ 0.05), as shown in Table S6.

### 3.5 Lipidome–Metabolites–AD

**FIGURE 3:**
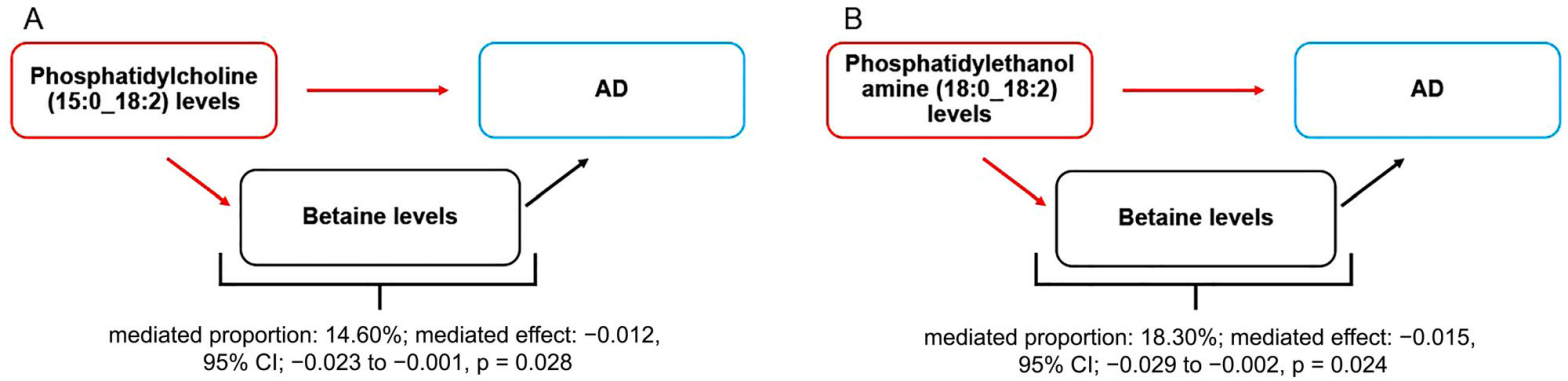
Schematic diagram of the metabolites‐mediated effect. (A) The mediated effect of betaine levels between phosphatidylcholine (15:0_18:2) levels and AD. (B) The mediated effect of betaine levels between phosphatidylethanolamine (18:0_18:2) levels and AD. AD, Alzheimer's disease.

The mediation MR analysis showed that both PC (15:0_18:2) levels and PE (18:0_18:2) levels mediated the reduction of AD risk by lowering betaine levels. Betaine levels mediated 18.30% of the reduction of AD risk associated with PC (15:0_18:2) levels (mediated proportion: 18.30%; mediated effect: −0.015, 95% CI: −0.029 to −0.002, *p *= 0.024), and 14.60% of the reduction of AD risk associated with PE (18:0_18:2) levels (mediated proportion: 14.60%; mediated effect: −0.012, 95% CI: −0.023 to −0.001, *p *= 0.028), as shown in Figure 3.

### 3.6 Heterogeneity and Sensitivity Analysis

Cochran's Q showed heterogeneity in the results for PC (15:0_18:2) levels–betaine levels (*p *= 0.048), PE (18:0_18:2) levels–betaine levels (*p *= 0.013), and PE (18:0_18:2) levels–AD (*p *= 0.001), while the other results were not significantly heterogeneous (*p *≥ 0.05), as shown in Figure S2 and Table S7. Leave‐one‐out sensitivity analysis indicated that these MR results were robust, as shown in Figure S3.

## 4 Discussion

### 4.1 Research Background and Results

The pathogenesis of AD remains incompletely understood, involving numerous disciplines such as neuroscience, genetics, and cell biology, and is a research hotspot in the broader life science field (Scheltens et al. 2021). With the global population aging and the prevalence of AD increasing, the importance of its prevention and treatment has escalated (Cummings et al. 2019). Understanding the role of the lipidome and its metabolites in the pathogenesis of AD will guide the development of new drugs for the prevention or treatment of AD. To the best of our knowledge, this is the first MR analysis assessing the causal effects of the lipidome on AD and the mediated effects of metabolites. The results of this study demonstrated that both PC (15:0_18:2) and PE (18:0_18:2) mediated the reduction of AD risk by lowering betaine levels. It revealed a complex link between PC (15:0_18:2) and PE (18:0_18:2) with AD, providing new insights into the pathogenesis of AD.

### 4.2 Effects of Lipidome on AD

PC (15:0_18:2) refers to PC with valeric acid (15:0) at the Sn‐1 position and linoleic acid (18:2) at the Sn‐2 position. PC, an essential component of cell membranes, exhibits abnormal differences between normal brains and those of AD patients (Nitsch et al. 1992). A Dutch meta‐analysis revealed significantly lower circulating PC levels in AD patients compared to cognitively intact elderly individuals (Hedges *g* −0.86; 95% CI: −1.28–0.44) (de Wilde et al. 2017). Decreased PC synthesis or increased utilization reduces brain PC levels, resulting in fewer synapses in the brain by affecting neuronal function (Wurtman 2015). Previous studies have suggested that loss of synaptic function and reduced synapse number are central mechanisms in AD pathogenesis (Peng et al. 2022), which suggests that low PC levels may induce AD by affecting synapse numbers. These pieces of evidence point to PC as a potential protective factor for AD and low PC levels as a risk factor for AD.

Subsequent research has indicated both valeric acid and linoleic acid in PC (15:0_18:2) play active roles in AD. Valeric acid, a major constituent of *Valeriana wallichii* DC, demonstrates various beneficial properties, including anticancer, antidiabetic, antihypertensive, anti‐inflammatory, and immunomodulatory effects (Kumari et al. 2024). Although there are no direct reports on the benefits of valeric acid in AD, literature supports its ability to improve Parkinson's disease prognosis by inhibiting inflammation and oxidative stress (Jayaraj et al. 2020). Related studies have shown that valeric acid protected dopaminergic neurons in rats with Parkinson's disease by mitigating rotenone‐induced inflammation and oxidative stress, as well as inhibiting the overactivation of microglia and astrocytes (Jayaraj et al. 2020). Notably, hyperactivation of microglia and astrocytes is also associated with impaired β‐amyloid clearance in AD (Singh 2022; Al‐Ghraiybah et al. 2022). β‐Amyloid gradually aggregates into soluble oligomers and insoluble protofibrils and ultimately contributes to synaptic dysfunction, neuronal inflammation, and oxidative stress injury (Haass and Selkoe 2007). Furthermore, linoleic acid (ω6; 18:2), the most consumed polyunsaturated fatty acid in the human diet (Whelan and Fritsche 2013), exerts anti‐inflammatory and neuroprotective effects in neurodegenerative diseases (Alarcon‐Gil et al. 2022). Previous studies suggested linoleic acid could reverse brain inflammation induced by microglia hyperactivation (Tu et al. 2019). A subsequent animal study showed that a linoleic acid‐rich diet improved AD prognosis by reducing proinflammatory factors such as interleukin (IL)‐1β and IL‐6 while increasing anti‐inflammatory factors such as IL‐10 in AD mice (Ma et al. 2020). These pieces of evidence suggest that valeric acid and linoleic acid reduce AD risk by inhibiting microglia‐ and astrocyte‐mediated inflammation or oxidative stress, pointing to PC (15:0_18:2) as a potential protective factor for AD.

PE (18:0_18:2) refers to PE consisting of stearic acid (18:0) at the Sn‐1 position and linoleic acid (18:2) at the Sn‐2 position. PE, as a multifunctional phospholipid required for mammalian development, accounts for 15%–25% of total phospholipids in mammalian cells (Vance 2015) is involved in various cellular processes such as cell membrane fusion, oxidative phosphorylation, mitochondrial stability, and autophagy (Calzada et al. 2016). A lipid analysis revealed significantly reduced PE levels in all three cerebral cortex regions of AD patients, while similar phospholipid abnormalities were not observed in the remaining neurodegenerative diseases (Nitsch et al. 1992). Another clinical study in Illinois demonstrated that low PE levels accelerated the median time for mild cognitive impairment to progress to AD by twofold, indicating that PE may be a biomarker for predicting the conversion of mild cognitive impairment to AD (Llano and Devanarayan 2021). These findings indicate PE as a potential protective factor for AD, with low PE levels posing a risk factor for AD.

In addition to PE itself, stearic acid at the Sn‐1 position and linoleic acid at the Sn‐2 position have also been linked to reduced AD risk. As mentioned earlier, linoleic acid improves AD prognosis by inhibiting the inflammatory response mediated by microglia hyperactivation (Tu et al. 2019; Ma et al. 2020). Stearic acid, a common saturated fatty acid, is considered a mitochondrial regulator (Senyilmaz et al. 2015). A study conducted at the University of California revealed that compared to non‐AD patients, AD patients exhibited a significant 27% reduction in the concentration of stearic acid in the prefrontal cortex, while the concentration of stearic acid in total brain phospholipids decreased by 26% (Otoki et al. 2021). Another study from the United Kingdom reported similar results, confirming a significant decrease in stearic acid in the frontal and temporal cortices of AD patients through large‐sample analysis (Fraser et al. 2010). These pieces of evidence support a close association between stearic acid deficiency and AD. In addition, the role of stearic acid in neurodegenerative diseases such as AD may be related to the regulation of mitochondria (Beal 1998). In advanced AD, loss of mitochondrial function affects APP expression and processing, exacerbating β‐amyloid accumulation (Swerdlow et al. 2014). However, stearic acid protects cortical neurons from lipid peroxidation by modulating mitochondrial function in a dose‐dependent manner (Wang et al. 2007), thus delaying the progression of neurodegenerative diseases (Bajracharya et al. 2019). These pieces of evidence suggest that PE, stearic acid, and linoleic acid all play active roles in AD, pointing to PE (18:0_18:2) as a potential protective factor for AD.

### 4.3 Effects of Metabolites on AD

Betaine is an important metabolite whose role in AD may be related to transmethylation to form dimethylglycine and to catalyze methionine formation (Eklund et al. 2005, Lever et al. 2017). First, methionine is a sulfur‐containing essential amino acid that plays a crucial role in AD (Alachkar et al. 2022). An animal study from China demonstrated that excessive methionine intake resulted in hippocampal and cortical damage and increased expression of APP and β‐secretase 1 in mice, leading to AD‐like symptoms (Pi et al. 2021). Another US animal study showed that methionine mediated neurological damage in AD mice by promoting microglia activation and enhancing neuroinflammation (Alachkar et al. 2022). Second, dimethylglycine, a potent reactive oxygen species (ROS) generator, stimulates ROS production in mitochondria (Mailloux et al. 2016). Current research suggests that ROS are associated with age‐ and disease‐dependent mitochondrial dysfunction (Tönnies and Trushina 2017). It directly affects synaptic function and neurotransmission in neurons and may ultimately lead to cognitive dysfunction (Tönnies and Trushina 2017). These findings support the association of methionine and dimethylglycine with an increased risk of AD, pointing to betaine as a potential risk factor for AD.

### 4.4 Effects of Lipid‐Regulated Metabolites on AD

The MR analysis revealed that PC (15:0_18:2) mediated the reduction of AD risk by lowering betaine levels, which accounted for 18.30% of the PC (15:0_18:2)‐associated reduction of AD risk. As the free fatty acids at the Sn‐1 and Sn‐2 positions of PC (15:0_18:2), valeric acid, and linoleic acid inhibited microglia‐ and astrocyte‐mediated inflammation or oxidative stress (Jayaraj et al. 2020; Alarcon‐Gil et al. 2022). Conversely, betaine enhanced microglia activation and neuroinflammatory injury in AD mice by catalyzing methionine formation (Alachkar et al. 2022; Pi et al. 2021). Therefore, we hypothesized that PC (15:0_18:2) may inhibit microglia activation‐mediated inflammatory responses by reducing betaine levels, thereby reducing AD risk.

The MR analysis also indicated that PE (18:0_18:2) mediated the reduction of AD risk by lowering betaine levels, which accounted for 14.60% of the PE (18:0_18:2)‐associated reduction of AD risk. As a free fatty acid at the Sn‐1 position of PC (15:0_18:2), stearic acid reduced the accumulation of β‐amyloid and lipid peroxidation by modulating mitochondrial function (Swerdlow et al. 2014; Wang et al. 2007). Conversely, betaine promoted β‐amyloid expression by catalyzing methionine formation and stimulated ROS production in mitochondria by conversion to dimethylglycine (Mailloux et al. 2016). Accordingly, we hypothesized that PE (18:0_18:2) may inhibit β‐amyloid formation and neuro‐oxidative damage by lowering betaine levels, thereby reducing AD risk.

However, it is noteworthy that in our MR analysis, the effect sizes of PC (15:0_18:2)‐AD and PE (18:0_18:2)‐AD were both small, with ORs of 0.926 and 0.928, respectively. This suggests that PC (15:0_18:2) and PE (18:0_18:2) were only associated with a 7.4% and 7.2% reduction in the risk of AD. Moreover, betaine, a mediated metabolite, could only account for 18.3% of the effect of PC (15:0_18:2) on AD and 14.6% of the effect of PE (18:0_18:2) on AD. This means that the PC (15:0_18:2)–betaine and PE (18:0_18:2)–betaine pathways only reduced the risk of AD by 1.4% and 1.1%, respectively. Although both of these mediated pathways were statistically significant, the effect sizes were extremely small, with probably no clinical relevance. Specifically, the magnitude of these mediated effects suggests that their impact on AD risk is likely limited and, in particular, may fail to be significant in clinical practice. Thus, although these results provide clues for further research, the clinical applicability of these effects may be limited, and future studies may need to explore more significant effects or other potential influences.

Furthermore, the precise mechanism by which betaine levels are influenced by PC and PE remains unclear, as this is not a known metabolic pathway. Therefore, PC and PE levels may covariate with betaine levels rather than an absolute causal relationship. We hypothesize that this covariation is related to “competition for choline.” Choline is a precursor substance for the synthesis of PC and betaine, and it can be oxidized either by the cytidine diphosphate–choline pathway to form PC or by choline dehydrogenase to form betaine. Choline plays an essential role in PC synthesis, and approximately 70% of PC in the human body is synthesized through choline (Jacobs et al. 2010). When choline is used excessively for PC synthesis, less choline is available for betaine, which may indirectly lead to lower betaine levels. Moreover, there is a certain interconversion and dynamic balance between PC and PE. PE produces 30% of the body's PC by phosphatidylethanolamine N‐methyltransferase (PETM), which also produces PE by phosphatidylserine decarboxylase and phosphatidylserine synthetase (Jacobs et al. 2010; Li and Vance 2008). Thus, when excess choline is used to synthesize PC, less PC is synthesized by PETM, which allows PE to be retained. Conversely, the consumption of choline leads to a decrease in the substrate and level of betaine. Ultimately, this covariation may lead to a situation where PC and PE levels increase while betaine levels decrease. The covariant relationship between PC, PE, and betaine is shown in Figure S4.

### 4.5 Limitations and Prospects

Although this study provides genetic insights into the analysis of lipidome–metabolites–AD, several limitations must be acknowledged. First, although sensitivity analysis showed the MR analysis results were robust, there was heterogeneity in three sets of analysis, which may increase the potential risk of bias. Second, since the lipidome, metabolites, and AD datasets included were all from the European population, the results of this study lack racial universality and should be cautiously applied to other races. Third, the lipidome dataset was derived from a cohort study involving individuals aged 45–66 years, a demographic considerably younger than the typical age range for AD, which may diminish the accuracy of causal inference. Fourth, PC (15:0_18:2)–betaine and PE (18:0_18:2)–betaine pathways only reduced the risk of AD by 1.4% and 1.1%, respectively. Although both mediated pathways mentioned above are statistically significant, the effect size is extremely small and may not be clinically relevant. Fifth, the causal effects of PC and PE on betaine levels are not strong based on known metabolic pathways. It suggests that the lipidome‐metabolite causal effects found in this MR analysis may be due to extreme covariation of multiple factors resulting in overfitting. Sixth, although this study revealed two pathways of lipidome–metabolites–AD, the biological mechanisms underlying each pathway formation remain unclear.

We expect future studies to address these limitations and continually improve: first, based on continuously enriching genomic data, MR studies in different races should be promoted to understand the roles of lipidome–metabolites–AD in different races. Second, genomic studies focusing on lipids within age groups characterized by a high incidence of AD need to be conducted. These studies aim to generate a dataset that enhances the precision of causal inference within the lipidome–metabolites–AD pathway. Third, multiple centers and large sample clinical studies are needed to validate our findings and re‐evaluate the clinical significance of the above‐mentioned lipidome–metabolites–AD pathways. Fourth, the lipidome–metabolite–AD pathways should be verified through animal experiments to explore the biological mechanisms underlying each pathway formation, thus providing strong biological evidence for this study.

## 5 Conclusion

The MR analysis revealed the pathways through which PC (15:0_18:2) and PE (18:0_18:2) reduce the risk of AD by lowering betaine levels. However, further studies are needed to validate these pathways and explore the biological mechanisms.

## Author Contributions

**Yunfeng Yu**: conceptualization, writing ‐ original draft, supervision. **Juan Deng**: writing ‐ original draft, formal analysis. **Xinyu Yang**: writing ‐ original draft, data curation, methodology. **Jingyi Wu**: writing ‐ original draft. **Rong Yu**: writing ‐ review and editing. **Chenlu Guo**: conceptualization, writing ‐ review and editing, supervision.

## Ethics Statement

The present MR analysis was based on summary data from previous studies obtained from public databases that had gained relevant, informed consent and ethics approval. No ethical permit is required for the secondary analysis of summary data.

## Consent

The authors have nothing to report.

## Conflicts of Interest

The authors declare no conflicts of interest.

### Peer Review

The peer review history for this article is available at https://publons.com/publon/10.1002/brb3.70352.

## Supporting information



Supporting Information

Supporting Information

Supporting Information

Supporting Information

Supporting Information

## Data Availability

The data that supports the findings of this study are available in the Supporting Information of this article.
