# Supplementary figures and images for: Association of the Lipidome With Alzheimer's Disease and the Mediated Effect of Metabolites: A Two‐Step Mendelian Randomization Study
Source: Brain Behav. 2025 Feb 19;15(2):e70352. doi: 10.1002/brb3.70352 (PMC11839762; doi:10.1002/brb3.70352)

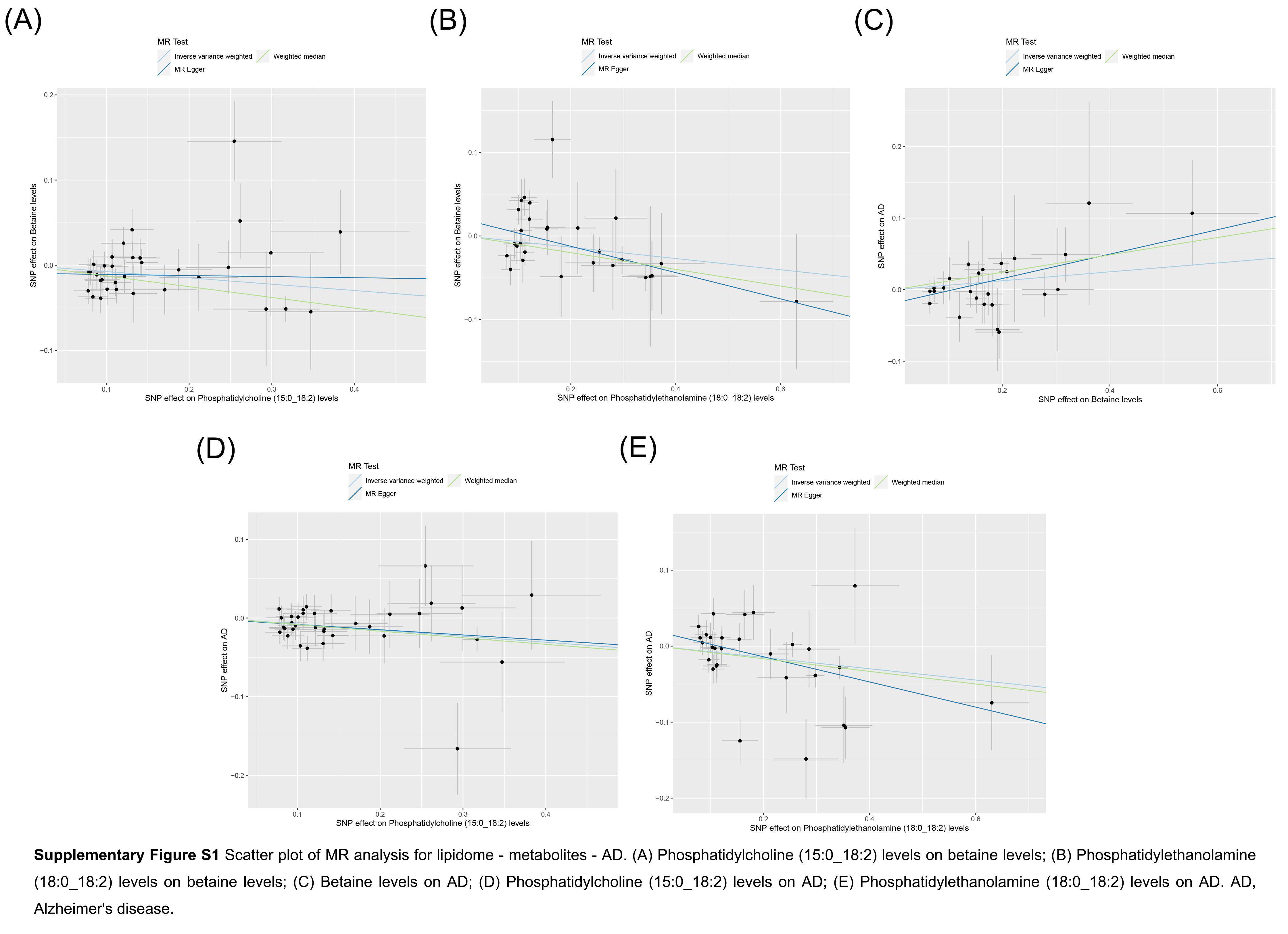

Supplement: Supplementary file 1 — Supporting Information [file BRB3-15-e70352-s003.jpg]

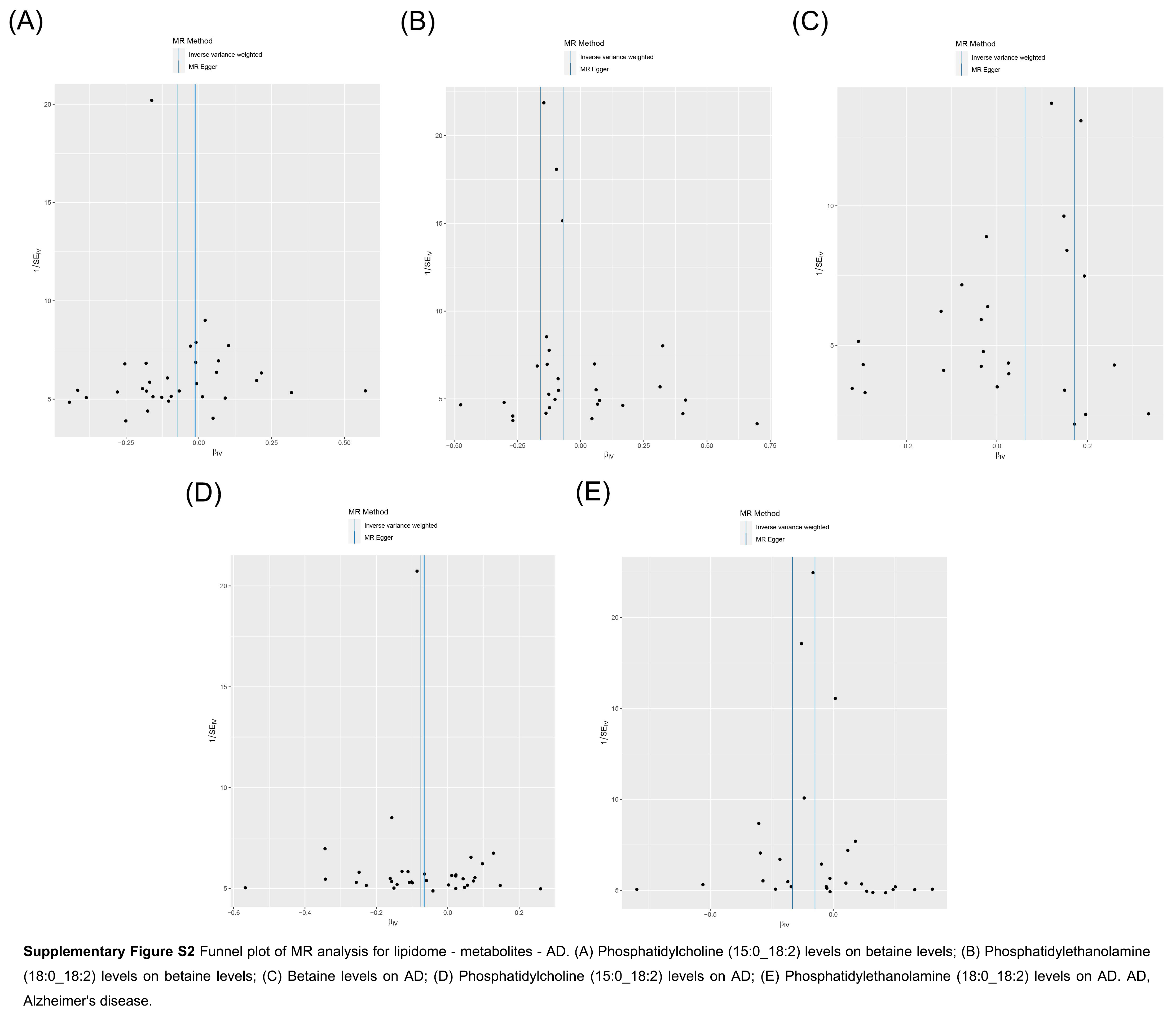

Supplement: Supplementary file 2 — Supporting Information [file BRB3-15-e70352-s002.jpg]

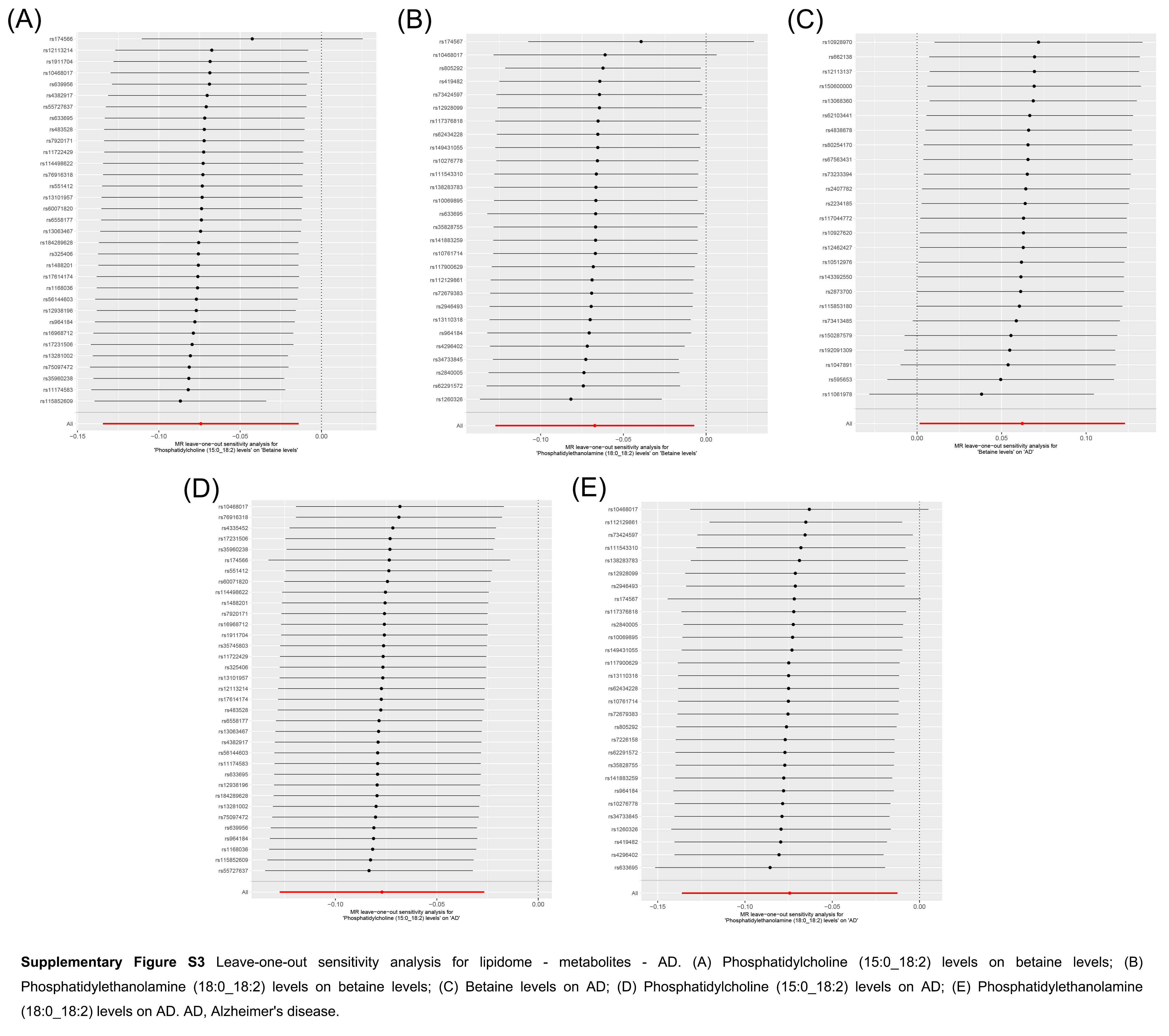

Supplement: Supplementary file 3 — Supporting Information [file BRB3-15-e70352-s004.jpg]

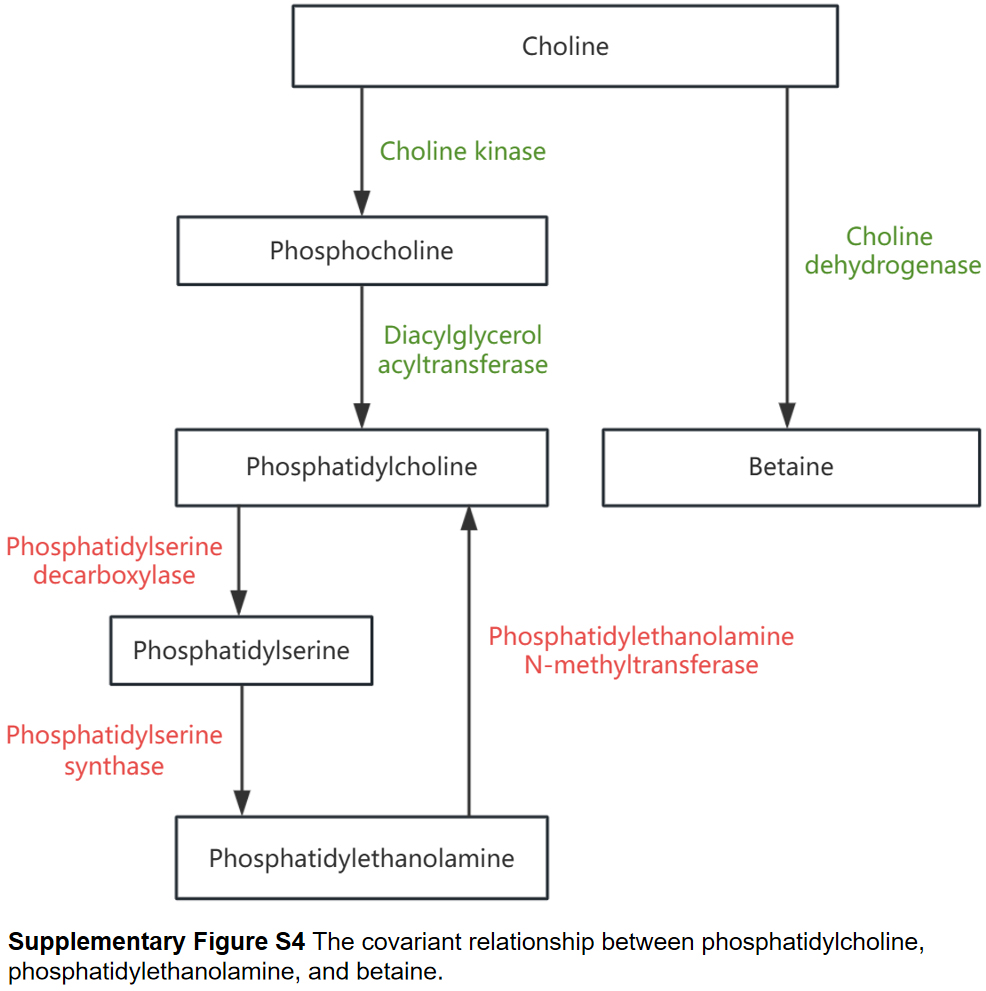

Supplement: Supplementary file 4 — Supporting Information [file BRB3-15-e70352-s001.jpg]
